# Barriers and facilitators to supporting women with postnatal depression and anxiety: A qualitative study of maternal and child health nurses’ experiences

**DOI:** 10.1111/jocn.16252

**Published:** 2022-02-14

**Authors:** Noushin Arefadib, Touran Shafiei, Amanda Cooklin

**Affiliations:** ^1^ Judith Lumley Centre School of Nursing and Midwifery La Trobe University Bundoora Victoria Australia

**Keywords:** Maternal child health, postnatal anxiety, postnatal depression, Public health nursing, qualitative study

## Abstract

**Aims and objectives:**

To explore maternal and child health nurses’ experiences of supporting women with postnatal depression and anxiety and the factors which impact these.

**Background:**

Maternal and child health nurses play a key role in identifying women with postnatal depression and anxiety and facilitating their access to appropriate supports. Understanding how nurses carryout this work, and the conditions which impact their ability to do so, is critical to the development of service delivery frameworks that can facilitate optimal outcomes for women and their families. Despite this, little is known about this subject.

**Design:**

A qualitative descriptive study.

**Methods:**

Participants were maternal and child health nurses practicing for at least six months and regularly seeing new mothers in Victoria, Australia. Twelve nurses were interviewed. Thematic analysis was conducted to identify patterns across our data. Qualitative content analysis was used to identify issues which were most emphasised by nurses. Reporting complies with the COREQ checklist.

**Findings:**

Three overarching themes were identified. Theme one pertained to steps taken by nurses following the identification of depression or anxiety symptoms and the shared challenges they encountered. Theme two concerned nurses’ experiences of supporting women who required acute mental health interventions and the systemic barriers they faced. Finally, theme three related to how the existing service delivery model could be improved to better support nurses in their work.

**Conclusions:**

The complex system within which nurses operate presents barriers that can impede their ability to respond to women with postnatal mental health issues. There is a need for service delivery frameworks that better support nurses and facilitates equitable access to mental healthcare.

**Relevance to clinical practice:**

Facilitating equitable access to all perinatal mental health services and interventions must be at the heart of all future policy, funding and service delivery frameworks.

**What does this paper contribute to the wider global clinical community?**

- Maternal and child health nurses hold a significant amount of responsibility in identifying women with symptoms of postnatal depression and anxiety and facilitating their timely access to appropriate supports.
- Understanding the factors which impede or facilitate nurses’ ability to carry out this critical work is central to the development of service delivery models that are evidence‐based and designed to facilitate optimal outcomes for women and their families.
- This study brings awareness to the professional needs of maternal and child health nurses in being able to effectively carryout their role, as identified by them.

## 1 INTRODUCTION

Postnatal depression and/or anxiety (PNDA) impacts between 10% and 20% of all mothers (Falah‐Hassani et al., 2017; Woody et al., 2017). Global evidence regarding the adverse and potentially long‐term impact of untreated PNDA on maternal and child outcomes is well established (Slomian et al., 2019). Moreover, recent studies from the Unites States (Luca et al., 2020), Australia (PricewaterhouseCoopers, 2014, 2019) and the UK (Bauer et al., 2016) have identified a significant economic burden associated with undetected and untreated PNDA, resulting from ongoing healthcare costs and lost income. Conversely, systematic reviews of global studies, including those set in Australia, Canada and across Europe and Asia (Dennis & Chung‐Lee, 2006; Dennis & Hodnett, 2007; Lumley et al., 2004) indicate that timely identification of PNDA through universal screening, followed by the facilitation of access to evidence‐based interventions (e.g. peer support and cognitive behavioural therapy) is cost‐effective, perceived by women as beneficial, and can significantly reduce PNDA symptoms and mitigate negative outcomes (Hadfield & Wittkowski, 2017).

Despite this, an alarmingly low proportion of women seek treatment (Dennis & Chung‐Lee, 2006; Fonseca et al., 2015; Henshaw et al., 2013; Holt et al., 2017). In a cross‐sectional study involving 656 Portuguese women, Fonseca et al. (2015) found that approximately 80% of women who screened positive for perinatal depressive disorders did not seek any professional supports. Similarly, in an Australian randomised controlled trial, Holt et al. (2017) found that 65% of women who screened positive for PND while receiving standard care, did not seek any form of treatment. Systematic reviews indicate that women's help‐seeking behaviours are influenced by similar factors around the world (Dennis & Chung‐Lee, 2006; Hadfield & Wittkowski, 2017). These encompass, but are not limited to, structural factors (e.g. screening practices among healthcare providers and availability of appropriate interventions); social factors (e.g. cultural stigma associated with mental illness and use of mental health services); personal factors (e.g. treatment preferences by women and views towards pharmacological treatments) and interpersonal factors which largely pertain to the provider‐maternal relationship (e.g. women's perceived absence of judgement by the health worker) (Dennis & Chung‐Lee, 2006; Hadfield & Wittkowski, 2017; Jones, 2019). To that end, Dennis and Chung‐Lee (2006) maintain that healthcare professionals play a pivotal part in either impeding or facilitating women's access to appropriate PNDA supports.

In Victoria, Australia, identifying women with PNDA and facilitating their access to appropriate supports is largely the responsibility of dual registered nurse/midwives known as maternal and child health nurses (MCHNs) (Departmnet of Health & Human Services, 2019). Maternal and child health nurses provide free universal care to all women with children aged birth to six years through the provision of ten (or more if required) Key ages and stages visits with a focus on prevention, early identification and intervention for a host of maternal and child health issues, including PNDA (Departmnet of Health & Human Services, 2019). With roughly 80% of all Victorian mothers visiting an MCHN approximately seven times in the first 12 months postpartum (Victorian State Government, 2019), MCHNs are exceptionally well‐placed to identify and support women with PNDA. The MCHN role is comparable with that of the Plunket nurse in New Zealand (Honey & Westbrooke, 2016), Public Health Nurse in Ireland and Norway (Glavin & Leahy‐Warren, 2013) and Health Visitors in the UK (Lowenhoff et al., 2017). Throughout this paper, the term ‘MCHN’ will be used to describe all nurses and midwives (discussed in the literature) whose role is comparable to that of Victorian MCHNs.

Victorian maternal and child health practice guidelines (Departmnet of Health & Human Services, 2019) recommend that MCHNs screen all women for PNDA during the fourth week visit by performing a psychosocial assessment (i.e. enquiring about known psychosocial risk factors such as a history of mental illness and family violence) and, where indicated, administering the Edinburgh Postnatal Depression Scale (Cox et al., 1987). Additionally, the guidelines recommend that MCHNs inform women of available support and referral options and facilitate access to these once a ‘positive’ identification has been made. However, the lack of routinely collected data pertaining to how MCHNs identify and support women with PNDA means that our understanding of this critical issue is limited.

This study is the second phase of a two‐phased mixed methods study which aimed to generate new evidence to address this gap. Phase one was a cross‐sectional survey of all MCHNs practicing in Victoria and examined MCHNs’ knowledge, attitudes and practices relating to the screening and management of PNDA (2019–2020). The aim of the present study was to gain a deeper and more nuanced understanding of MCHNs’ experiences of screening and supporting women following the identification of PNDA symptoms. This manuscript reports exclusively on the study's findings pertaining to the actions which took place once a positive PNDA identification was made, as well as the factors which influenced these. While MCHNs’ screening and management practices are intrinsically connected and cannot be separated from one another in practice, the factors which influence each are overall distinct (Arefadib et al., 2021). In a recent scoping review of the range and nature of primary research on PNDA screening and management by MCHNs, Arefadib et al. (2021) found that MCHNs’ PNDA screening practices were largely influenced by how they perceived their role, their attitude towards PNDA and screening tools, as well as their knowledge and training. However, MCHNs’ management of PNDA, following identification, was influenced by availability of formal care pathways, availability and access to services, continuity of care and collaboration and cohesion between service providers. To the best of our knowledge, no previous studies have provided an in‐depth assessment of MCHNs experiences following the identification of PNDA symptoms.

Understanding the nuances of how MCHNs support women with PNDA, and the conditions which impact their caregiving capacity, is critical to the development of service delivery models that are evidence‐based and designed to facilitate optimal outcomes for women and their families. Moreover, it brings awareness to the professional needs of MCHNs in being able to effectively carryout their role, as identified by them. As such, this study aimed to 1) describe what actions MCHNs took once they had identified PNDA; and 2) understand the conditions/factors which impede or strengthen their ability to support women with PNDA effectively.

## 2 METHODS

### 2.1 Design

A qualitative descriptive design was used. Qualitative descriptive studies are committed to examining events or experiences in their natural state and allow a comprehensive presentation of a phenomenon in the everyday language of participants (Sandelowski, 2000). To that end, the intent of this approach is to convey facts, and the meanings attributed to these facts, as defined by participants. Qualitative descriptive studies are founded on the general principles of naturalistic enquiry (Colorafi & Evans, 2016; Lincoln & Guba, 1985), which centre on the concept of truth and require the researcher to observe events in their natural state without attempting to manipulate how events unfold. The study is reported in compliance with the Consolidated criteria for Reporting Qualitative Research (COREQ) (Tong et al., 2007) [Supplementary file].

### 2.2 Participants and recruitment

Eligible participants were MCHNs who (1) undertook key ages and stages visits with new mothers as a primary component of their role (2) had been practicing in Victoria as an MCHN for a minimum of 6 months and (3) had participated in the phase one survey and agreed to be re‐contacted for participation in the present study. The final survey question in phase one asked if MCHNs would be willing to participate in a follow‐up interview. Those who said ‘yes’ were invited to provide their contact information (either phone or email address). A total of 62 MCHNs agreed to be interviewed, of which purposive sampling was used to ensure variability in participants’ attitudes towards screening, age, years of professional experience, geographic area (metropolitan versus regional) and concentration of socioeconomic disadvantage in the local government area in which they practiced. Data from the Australian Bureau of Statistics’ Socio‐Economic Indexes for Areas (Australian Bureau of Statistics, 2016) used as an indicator of each area's relative socioeconomic disadvantage (low versus high), compared with other local government areas in Victoria. The Australian Bureau of Statistics determine socioeconomic advantage and disadvantage by drawing on variables including income, education, employment, occupation and housing (Australian Bureau of Statistics, 2016).

Twelve MCHNs were initially invited to participate via email. Of these, one informed us that she no longer worked as an MCH, and three were uncontactable, at which point four more MCHNs were invited to participate. The authors agreed that data saturation had occurred when the data acquired during the interviews became redundant and that additional interviews would add little to our understanding of the study subject. The authors perceived saturation after ten interviews; however, two additional interviews were carried out for confirmation.

### 2.3 Data collection

Between March and May, 2021, interviews with semi‐structured questions were held via Zoom, a cloud‐based videoconferencing service (Inc, 2016). All interviews were conducted by the first author and lasted between 30 and 45 min. Verbal consent to participate and for the interview to be audio‐recorded was obtained prior to the commencement of all interviews. An interview guide was created to address gaps in our understanding regarding how MCHNs’ managed PNDA. Questions were in part informed by the survey results of phase one, which highlighted these gaps. For example, we realised from the survey findings that we still had a very limited understanding of how MCHNs met the needs of women who required critical mental health supports. To address this gap, a question regarding this critical issue was included in the interview guide. Overall, questions were designed to strengthen our understanding of MCHNs PNDA management practices, and the conditions which influenced them. Prior to the study, pilot interviews were held with three MCHNs (not included in the study sample) to assess the suitability, wording and order of the questions to be asked, along with any issues which may have prevented us from obtaining quality data. This resulted in minor changes to the wording and ordering of some questions. Interview questions are presented in Table 1. All interview and participant data were stored on password protected computer files, operated by La Trobe University. All data will be securely stored for a minimum of 5 years following publication.

**TABLE 1:** Interview guide

Can you tell me about what you usually do once you’ve identified a mum with PNDA symptoms?
What sorts of things are likely to influence your decision?
What do you typically do when you identify a mum who needs critical/urgent care?
What would make it easier for you to support mothers with PNDA?
Is there anything that we haven’t discussed today that you feel is important and should be included in this conversation and certainly in this study?

### 2.4 Analysis

All interviews were anonymised and later transcribed verbatim. To facilitate authentic engagement with the data, the initial five interviews were transcribed by the first author; all subsequent interviews were transcribed by a professional transcription service. Data were analysed using thematic and qualitative content analysis. First, thematic analysis, using Braun and Clarke's (Braun & Clarke, 2006) six‐step process, was conducted to systematically identify and organise patterns across our data. This involved coding the data in an iterative process where all three members of the research team initially coded three interviews separately and discussed any variations until consensus on codes and respective meanings were reached. Given the limited discrepancy between how the three authors assigned codes and meaning to the data, the remaining nine transcripts were coded by the first author who routinely sought feedback from the second and third authors during this process. All final codes were independently assessed by the second and third authors.

Similar codes were grouped together, and themes were identified and defined in a joint process by all authors. Qualitative content analysis (Sandelowski, 2000) was then used to confirm and better understand each theme by counting the number of times each response (code) was identified. Counting the number of repetitions allowed us to identify which issues were most emphasised by MCHNs. Data are presented in themes and frequency (Table 2).

**TABLE 2:** Themes, sub‐themes and frequencies

Theme	Subtheme	Frequency
What happens next? referral power, availability and access	Referral power: an uneven distribution between MCHNs and GPs	10
	Availability and access—the role of place and socioeconomic status	12
In case of emergency	Mental health crisis supports: what are the options?	12
	Barriers to facilitating access to crisis and intensive supports	11
Going forward—how MCHNs can be better supported to facilitate women’s	Not just nurses: the need for a multidisciplinary team and co‐location of services	8
	More time and resources, not more training	9
	More direct referral power	5

### 2.5 Establishing credibility, transferability, and dependability

We followed Lincoln and Guba's (1985) strategies for strengthening trustworthiness in qualitative research, which includes criteria for credibility, transferability and dependability. Credibility relates to how accurately findings reflect the truth and are based on the assertions of participants (Korstjens & Moser, 2018; Lincoln & Guba, 1985). To strengthen our study's credibility, we used investigator triangulation, persistent observation of the data and relevant literature and prolonged engagement with the data (Korstjens & Moser, 2018). Transferability pertains to how accurately the findings can be transferred to other settings (Korstjens & Moser, 2018). To increase transferability, we provided context to participant's experiences, so that they would become meaningful to readers unfamiliar with components of the study (e.g. the MCH service). Dependability and confirmability relate to the extent to which the research and reporting process are systematic, transparent and accurate (Korstjens & Moser, 2018). To increase our study's dependability and confirmability, we maintained a detailed audit trail for each stage of the study.

### 2.6 Ethics considerations

This study was approved by the La Trobe University, Science Health & Engineering College Low Risk Human Ethics Committee (reference HEC18512) and the Department of Health and Human Services (DHHS), Centre for Evaluation and Research.

## 3 FINDINGS

### 3.1 Participant characteristics

Our sample consisted of 12 MCHNs, working across 11 local government areas in regional (*n* = 4) and metropolitan (*n* = 8) Victoria. Participants’ characteristics are reported in Table 3. Participants were all female and predominantly worked part‐time. Seven MCHNs worked in communities with low socioeconomic status, and half had indicated (in study one) that they were able to identify PNDA without screening for it (i.e. without the use of a tool such as the Edinburgh Postnatal Depression Scale).

**TABLE 3:** Demographic information of participants (*n* = 12)

	*n* (%)
Age	
45–54	6 (50)
55–64	4 (33)
*≥*65	2 (17)
Years of experience as an MCHN	
3–9	2 (17)
10–20	7 (58)
>20	3 (25)
Role	
Universal MCHN only	6 (50)
Enhanced & Universal MCHN	5 (42)
Enhanced MCHN only	1 (8)
Employment hours	
Part‐time	9 (75)
Full‐time	2 (17)
Casual/Relief	1 (8)
Gender	
Female	12 (100)
Remoteness area	
Metro	8 (67)
Regional	4 (33)
Level of disadvantage in local government area	
High disadvantage (1–5)	7 (58)
Low disadvantage (6–10)	5 (42)

### 3.2 Themes

Three overarching themes were identified. Theme one, ‘what happens next? referral power, availability and access’ comprised two sub‐themes relating to MCHNs’ typical steps following the identification of PNDA and the factors which influenced these decisions. Theme two, ‘in case of emergency’ included two sub‐themes pertaining to how MCHNs facilitate women's access to acute mental healthcare and the challenges that they face when doing so. Finally, theme three, ‘going forward—how MCHNs can be better supported in facilitating women's access to appropriate mental healthcare’ comprised three sub‐themes relating to how MCHNs could be better supported in continuing their important work. Representative quotes from a range of MCHNs have been included, along with brackets containing each MCHNs’ unique de‐identified code and the geographic area in which they work—MCHNs working in a regional area are identified with ‘R’ and those working in metropolitan areas are identified with ‘M’ (e.g. MCHN 1, M).

#### 3.2.1 Theme 1: What happens next? referral ‘power’, availability and access

What happened after MCHNs had identified PNDA symptoms was overall determined by two factors: (1) who/where the MCHN had the ‘power’ to refer to; and (2) what services were simultaneously available and accessible. Findings pertaining to each are presented in two respective sub‐themes.

##### Referral power: an uneven distribution between MCHNs and GPs

All MCHNs said that, unless they had identified an immediate risk of harm to mother and/or infant, their first step following identification of PNDA symptoms, was typically to refer women to a GP. Most MCHNs (*n* = 10) said that referring to a GP was their first step because they were extremely limited in who/where they could refer to, with most mental health services (e.g. psychologists) and facilities (e.g. mother‐baby units) requiring a referral from a GP as a condition of access. Furthermore, MCHNs expressed that for women to receive financial reimbursement associated with the cost of accessing most mental health services, they were required to attain a ‘Mental Health Treatment Plan’ (MHTP), which only a physician had the authority to complete.

All we can do sometimes is (refer to GP)… If they (mothers) need more than that we're just going to hope their GP will do more… for us to rely on them to do the right thing by the client is hard. (MCHN 6, R).

Most MCHNs (*n* = 10) identified several issues with being so heavily reliant on GPs to support women with PNDA. The most cited issue (*n* = 7) was that the quality of care that women with PNDA received was directly proportional to how much interest each GP had in PNDA, how well‐informed the GP was (e.g. symptomology, availability of local supports), and how ‘seriously’ they took PNDA. MCHNs described how this resulted in noticeable practice variations and inconsistencies between how each GP treated PNDA. They shared that while some GPs provided ample information regarding a range of treatment options (e.g. medications and available support services), some solely offered antidepressants, and some even dismissed women's experience of PNDA.

I’ve had a mum I’ve sent off to the GP, and the GP rang me back and said, what do you want me to do about it? A lot of our GPs will say, you're a new mum, of course you're anxious. They downplay both at times (depression and anxiety), but more the anxiety. (MCHN 9, M).

… She went to see the GP. Put her on medication. I said, ‘well, how about that referral for a counsellor for talking therapies?’ and she said, ‘oh, no, the GP just wants me to try the medication first. (MCHN 7, R).

I don't think the GPs have the time or the knowledge base to go that next step… you say, ‘Okay, did you talk to your GP about this?’ And she'll say, ‘they didn't ask me any questions, so I didn't bother telling them.’ So they've come back with no referral, no medication. (MCHN 10, R).

Four MCHNs also expressed that the prerequisite of having to see a GP before being able to use services served as a barrier to accessing supports, particularly for women who would benefit from it most—those struggling to stay on top of their daily activities, in addition to meeting the demands of parenthood. Furthermore, MCHNs viewed their limited referral power as a missed opportunity for facilitating women's access to supports at a time when they were likely to be most receptive to accessing it:

…they're being honest about their struggles, and you feel like this would be a great opportunity to refer them while they're receptive, and you're like, ‘yeah, head off to the GP.’ Do they go? Are they going to be comfortable to discuss these things? It's just extending the process, and they can fall through the cracks because when your mental health is really bad, you're often not in a place to go off to the GP and talk about it. (MCHN 12, M).

The more there is to do for women with anxiety and depression, the less gets done. They are not able to get themselves to various appointments. (MCHN 3, M)

Lack of communication from GPs after a referral made by MCHNs was identified as problematic by six MCHNs, who felt that this resulted in disjointed ongoing service provision. MCHNs gave examples of writing referral letters, along with completed Edinburgh Postnatal Depression Scale forms, but failing to receive any follow‐up communication from GPs. Three MCHNs felt that this lack of communication may have been the result of GPs not valuing the role of the MCH service.

I’ve written a few letters (to GPs) recently and not heard a thing back which is very disappointing. I don't know how important they (GP) see our role. Ignorance maybe. (MCHN 1, M).

You normally hear nothing from the GP, even in emergency situations I think it's just they're time poor, or they don't value our service. (MCHN 12, M)

##### Availability and access—the role of place and socioeconomic status

All MCHNs described how availability of, and access to, appropriate mental health supports were significantly impacted by three key and often overlapping, factors: women's geographic location, financial means and ethnicity (particularly English proficiency). Three MCHNs described how their local council provided additional funding specifically for supporting women in the postnatal period, including home visitation programs for mothers experiencing, or at risk of, PNDA and supported playgroups (in various languages). Additional funding also meant that MCHNs were able to offer extra appointments to women who needed it. The provisions of these added supports meant that women requiring PNDA supports were more likely to access appropriate and timely supports. All three MCHNs worked in three respective affluent local government areas, with very low concentrations of socioeconomic disadvantage.

We're fortunate to receive good council funding on top of the State Government funding. So, just our availability of appointments means we can bring anyone back more frequently, without taking away from something else. (MCHN 5, M).

Conversely, nine MCHNs (seven of whom worked in local government areas with high concentrations of socioeconomic disadvantage) said that the lack of adequate PNDA support options, combined with an inability to offer additional appointments (due to limited funding) was a significant problem. Not only did this issue impact new mothers (who were unable to access timely supports), but it also resulted in MCHNs having to carry the ethical and moral responsibility of supporting mothers with PNDA, when no other service was available to do so. With no alternative available to them, many MCHNs resorting to using their personal time to contact women for follow‐up and to ensure their safety. Fatigue, feeling emotionally and mentally overwhelmed and a sense of defeat was a broadly shared experience among these MCHNs.

It's important to know how much MCH nurses care. There comes a point where you've just got nothing else left to give and no other services that you can recommend (and) you're personally depleted. …if we could do it the way we wanted to do it instead of what we are left with because there's no funding, you'd do what really needed to be done… (MCHN 3, M).

I may, shock horror, contact the client if it's a Saturday, make sure they're all right. But then council are very clear to say, ‘Oh, well, if you say or do something then, you're not protected by us.’ (but) you can get emotionally involved…you care. (MCHN 2, M).

They (employer) don't want us to hold them but then give us the right supports or places to refer them to. Child protection won't even take half of them, so you're still holding them and that's a really big thing. (MCHN 1, M).

Lack of suitable PNDA support options for Culturally and Linguistically Diverse (CALD) women (particularly those with limited English) was identified by five MCHNs; while the lack of support options for women residing in regional areas was identified by all four MCHNs who worked across their four respective regional local government areas.

I think there's this silent epidemic in the health system of racism, cultural racism. And if you don't have good English, you can't bat for yourself, you're kind of screwed, really. (MCHN 2, M).

We are not (adequately resourced), because we're regional and we don't have the services. And, even less so out in the outer region of our shire. (MCHN 6, R)

Where services were available, most MCHNs described barriers to access, including long waitlists (*n* = 8), cost (*n* = 5) and distance/transportation issues (*n* = 3). MCHNs felt that issues relating to access predominantly impacted vulnerable and disadvantaged mothers who could not afford to pay for private treatment facilities, and/or costs associated with travelling to existing facilities.

A lot of them (mother‐baby units) are private, and most of our clients don't have private health cover, so they can't afford it and public (mother‐baby) beds are not available… there's huge waiting lists. It's bad news, which adds to depression, family depression. (MCHN 4, M).

Some of the most vulnerable ones are the ones at the lower socioeconomic and they can't go out of town, they can't go far… (services) can be two hours for them. So, if they're really unwell to get them to go anywhere is really difficult. And if you've got a mental health issue a waitlist is no good. (MCHN 6, R).

A private psychologist costs… your mental health plan doesn't cover it. And that's a problem for my (CALD) clients. (MCHN 3, M)

#### 3.2.2 Theme 2: In case of emergency

The experience of identifying, and responding to an emergency, that is an immediate risk of harm (to mother and/or child) was shared by all twelve MCHNs. Findings pertaining to how MCHNs supported women through this experience, as well as the challenges they faced along the way, are presented in the following two sub‐themes.

##### Mental health crisis supports: what are the options?

MCHNs expressed that in instances where an immediate risk of harm (to mother and/or child) was identified, they had two main options available to them: (1) seven MCHNs said they would contact the Acute Community Intervention Service (ACIS)—an interdisciplinary team of professionals who assess risk and, where appropriate, respond to urgent requests for support in the event of a mental health crisis (Victorian Department of Health, 2014); and (2) five MCHNs said they would advise the woman to attend her closest hospital emergency department (ED), or call an ambulance to transport her to ED. While the ‘Mother Baby Unit’ (MBU) was also frequently identified (*n* = 6) as a suitable option for women experiencing acute PNDA, MCHNs advised that they did not have the ability to refer directly to this facility, which required a referral from a physician for access. For instances, where MCHNs had not identified an immediate risk of harm but believed that a woman required more immediate and intensive supports, existing options for this circumstance included referring to child protective services (*n* = 3) and/or to an enhanced MCHN (*n* = 7), who work with families experiencing multiple and concurrent complex challenges (e.g. family violence, drug and alcohol abuse).

##### Barriers to facilitating access to crisis and intensive supports

Most MCHNs (*n* = 11) identified a range of barriers associated with supporting women's access to these crises support options, the most frequent of which (*n* = 6) was the extremely high threshold for risk required for access to each.

Sometimes the nurses have assessed the client and have thought she's at considerable risk and ACIS has assessed them and disagreed. (MCHN 8, M)

…that's the only thing they're (ACIS) interested in; is your risk assessment and your information from your risk assessment. (MCHN 9, M)

They (Enhanced MCH) say, “is her mental health impacting her parenting ability?” Well, no, not at the moment. “Well, she's not in the enhanced criteria”. (MCHN 12, M)

Other identified barriers to facilitating access to immediate/crisis support options included limited availability and the long waitlists this resulted in (*n* = 5):

We did have a lady, very severe postnatal depression, and we couldn't even get her into the mental triage at the hospital. The nurse that was dealing with it, was blocked at every door… (MCHN 11, R).

There is a lack of services…there's never any beds in the mother baby units. (MCHN 3, M)

Slow response time was another identified barrier. This related to either waiting a long time for the ACIS team to arrive, or having to wait in ED for a long time before being assessed, and the adverse impact that this can have on access (*n* = 5):

I'll often have to call them (ACIS) and then the mother has to sit around for ages. We're in the office still to make sure they're in a safe place. We're still trying to keep going with our work. Sometimes it's after hours before they even respond. (MCHN 4, M).

I find people don't want to go into emergency because… there's still that stigma. A lot of them don't want people to know that they're not well. And because they're not losing a leg or dying… they're generally there for a while waiting. (MCHN 6, R).

The final identified barrier pertained to limited integrated service delivery. This was characterised by lack of communication between crisis support services and the MCH service, particularly regarding long‐term support plans set in place prior to discharge (*n* = 2).

…we ended up calling an ambulance to the hospital. They discharged the mum within three hours and not any services put in place. (MCHN 9, M)

#### 3.2.3 Theme 3: Going forward—how MCHNs can be better supported to facilitate women's access to appropriate mental healthcare

MCHNs identified numerous ways in which they could be better supported in ensuring that women experiencing PNDA can access appropriate and timely supports. These are presented in the following three sub‐themes.

##### Not just nurses: the need for a multidisciplinary team and co‐location of services

Most MCHNs (*n* = 8) working in both regional and metropolitan Victoria said that being able to effectively support mothers experiencing PNDA required a multidisciplinary approach, characterised by the co‐location of a team of professionals (e.g. psychologists, social workers, family support and domestic violence workers). MCHNs described the perceived benefits to this approach, including access to the expertise and support of other relevant professionals, with whom they could share the enormous responsibility of their work; as well as tackling some of the barriers to women accessing supports, such as distance, lack of transportation and long waitlists.

…a team of not just nurses but of counsellors, and family support workers… I think that's crucial so they can all work together because it's a tough gig. (MCHN 1, M)

Having a social worker sitting here next to me. Having those other people close by, that can help. (MCHN 11, R)

##### More time and resources, not more training

Lack of sufficient time to adequately support women experiencing PNDA, coupled with the long list of tasks that MCHNs were assigned with, was identified as a significant barrier to the provision of adequate care (*n* = 9). While MCHNs were not opposed to completing ongoing mental health‐related training, they felt training was redundant without the simultaneous provision of adequate time for effective engagement and support delivery, as well as suitable referral options.

I don't need hours and hours of training around this, unless someone's going to give me the time to do the stuff. (MCHN 2, M)

The problem isn't training us up, it's finding somebody to refer to afterwards. Because, yeah, we'll do the screening… but then you've got to find somewhere to send the client. (MCHN 9, M).

##### More direct referral power

The benefits of being able to refer women directly to appropriate mental health services, particularly in circumstances where there was urgency around the provision of care, were identified by five MCHNs.

… we can't refer to (the mother baby unit) directly, which would be amazing if we could. Because it's so hard to get someone like that to go somewhere, to get that referral—they're not in a state to do that when they're that bad. (MCHN 11, R).

… they (GPs) only see people for 10 minutes. It's not realistic. I know they're very protective of their role and don't like to give things up…but I don't think we should have to rely on them. (MCHN 6, R).

## 4 DISCUSSION

To the best of our knowledge, this is the first qualitative study to explicitly examine Victorian MCHNs’ experiences of supporting women with PNDA and the factors which enable or impede their ability to do so effectively. We identified three overarching themes: theme one pertained to the typical steps taken by MCHNs following the identification of PNDA and how place, socioeconomic status and MCHNs’ capacity to refer women directly to specialist services shaped these steps. The second theme concerned MCHNs’ experiences of supporting women who required acute mental health interventions and the systemic barriers they encountered in facilitating access to such supports. Finally, theme three pertained to how the existing service delivery model could be improved to better support MCHNs to continue their important work with greater ease and efficacy.

We found that MCHNs’ inability to directly refer women to specialist mental health services meant that they (and women) were largely dependent on GPs to facilitate women's access to appropriate psychological interventions, posing two fundamental issues. The first was that the requirement to attend another appointment at a different location posed a potential barrier to access for women whose PNDA symptoms made keeping up with daily tasks particularly challenging. This was concerning as it implied that those with more severe PNDA symptoms, and hence the greatest need for support, were potentially missing out on accessing much needed care. The assertion that women with PNDA are less likely to access support when faced with logistical barriers (e.g. travelling alone with an infant, lack of childcare and limited support) and the requirement to consult multiple professionals who are not co‐located, is consistent with evidence from a recent UK study (Ford et al., 2019) where similar to Australia, primary care is central to the provision of health services (Weller, 2006). In their study of 71 postpartum women in the UK, Ford et al. (2019) found that 42% of women who screened positive for PNDA did not see their GP for support, citing the logistical challenge of attending the appointment as a primary reason. Moreover, despite differences in the healthcare systems of the United States and Australia, similar findings were reported in an American study by Albaugh et al. (2018) who found that of the 647 women referred for perinatal mental health treatment, those who were offered home visits or appointments that were co‐located with their postpartum visit were four times more likely to access care.

The second issue with MCHNs overwhelming reliance on GPs was that the quality of care received by women (e.g. whether she was asked about her mental health, how validated she felt if a disclosure was made, what information she received, what treatment options were provided) was largely dependent on their GP’s knowledge of, and attitude towards PNDA. Evidence from a systematic review by Ford et al. (2016), which included studies from the United States, Australia, UK, Netherlands and Canada, supports this, showing that GPs treatment options for PNDA were significantly influenced by their attitude, training and knowledge of PNDA. Moreover, how women perceive their therapeutic relationship, particularly if they feel validated, listened to without judgement and are offered choice and control in all aspects of their care, is pivotal to how likely they are to access PNDA supports (Chew‐Graham et al., 2009; Dennis & Chung‐Lee, 2006; Hadfield & Wittkowski, 2017; Megnin‐Viggars et al., 2015). In their systematic review of the experiences of 585 women from the UK, Canada, Japan and Australia, who had sought support for PND, Hadfield and Wittkowski (2017) found that having health workers minimise their symptoms was a commonly shared experience, leading many to refrain from discussing this issue again in subsequent appointments. Megnin‐Viggars et al. (2015) reported similar findings in their systematic review of women's experiences in the UK indicating that women's access to postnatal mental health treatment was significantly hampered by their belief that their GP was unwilling to listen to, or was dismissive of, their psychological distress. Considering this evidence, we were concerned to find that many of the MCHNs in our study had encountered GPs who minimised women's PNDA symptoms and believed that depression and/or anxiety was a ‘normal’ response to the challenges associated with a newborn.

MCHNs also expressed concern regarding GPs’ proclivity to recommend pharmacological interventions without offering women alternative options, including talk‐therapy. Evidence suggests that women favour non‐pharmacological interventions to treat PNDA and that many avoid seeking help if they anticipate that medication will be the only treatment option offered (Buist et al., 2006; Chew‐Graham et al., 2009; Hadfield & Wittkowski, 2017). In a qualitative study which took place in 11 countries (eight European countries, Japan, Uganda and the United States), Oates et al. (2004) found that having someone to talk to and talk‐therapy were universally indicated as a treatment preference among women with PND. Our finding regarding MCHNs’ experiences of GPs’ promoting the use of antidepressants over other forms of interventions is consistent with international evidence (Britt et al., 2016; Buist et al., 2006; Chew‐Graham et al., 2009; Ford et al., 2016, 2019). In their systematic review, Ford et al. (2016) found that among GPs, antidepressants were the first and preferred option for treating PND (57%–92%). Similarly, in their UK study, Ford et al. (2019) found that of the women who had spoken with their GP about PNDA symptoms, 83% were offered antidepressants while only 29% were offered referral to therapy.

Our findings also indicate that scarcity of appropriate mental health referral options, including acute mental health services, was a challenge shared by many MCHNs. Moreover, where services were available, access was largely constrained due to barriers such as distance, limited transportation, cost and long waitlists. While issues pertaining to availability and access were widespread, our findings suggest that they may be particularly problematic in areas with greater socioeconomic disadvantage, rural/remote regions and communities with a large CALD/non‐English‐speaking population. Previous research supports this finding, indicating that vulnerable and disadvantaged women are less likely to access PNDA supports than their more advantaged counterparts due to a greater number of barriers to accessing care (e.g. cost, distance, limited transportation, language limitations and structural racism) (Falah‐Hassani et al., 2015; O’Mahony & Clark, 2018; Ogbo et al., 2018; Prady et al., 2021; Wright et al., 2018). In a recent study of the characteristics of mothers admitted to a mother‐baby unit in Auckland, New Zealand, Wright et al. (2018) identified significant disparities in equity of access, with older, more educated, white women being admitted for PND, while Pacific Island and Asian women were only admitted if they had a diagnosis of schizophrenia or bipolar disorder. This was in contrasts to the fact that Pacific Islander and Asian women had the greatest prevalence of PND in Auckland (Wright et al., 2018). Such findings are concerning given that low socioeconomic status, limited local language proficiency, being an immigrant (Falah‐Hassani et al., 2015; Ogbo et al., 2018) and living remotely (Bilszta et al., 2008; Mollard et al., 2016; Villegas et al., 2011) are all the risk factors for greater likelihood of PNDA.

Additionally, we found that the absence of a coordinated and multidisciplinary approach to responding to the needs of women with PNDA created a strong sense of duty on the part of MCHNs to support these women when no other service/professional was available to do so. Evidence suggests that facilitating the accommodation of care through flexible appointment times and giving women the opportunity to talk about their feelings in an unrushed manner are central to enabling women to disclose their PNDA symptoms and is associated with greater likelihood of engagement with supports (Dennis & Chung‐Lee, 2006; Hadfield & Wittkowski, 2017; Viveiros & Darling, 2018). Despite this, the inability to offer longer visits and/or additional appointments to women who required it most, was the most identified challenge by MCHNs in our study.

### 4.1 Strengths and limitations

This is the first study to explore in detail, Victorian MCHNs’ experiences of supporting women with PNDA, as well as the factors which hinder or facilitate their ability to carry out this important work effectively. Our purposeful sampling enabled diversity across a range of participant personal and professional characteristics. Moreover, analysis was carried out in line with Braun and Clarke’s (2006) six‐step process, and the study was reported in compliance with the COREQ checklist (Tong et al., 2007). Our study also has several limitations. It is possible that our findings are influenced by self‐selection bias given that those who participated in the study may have a special interest in perinatal mental health, and as such their views and practices do not accurately reflect those of all MCHNs. Additionally, despite reaching data saturation, our relatively small sample size means that it is possible that our findings are not transferable to all (approximately) 1300 MCHNs currently practicing in Victoria, and as such should be interpreted with caution. Finally, there are broader issues, such as negative public discourse and stigma surrounding mental health, as well as healthcare funding structures, which may influence some of the issues identified by MCHNs in this study. While these were not within the scope of our study, it is important to acknowledge and highlight them as topics for future research.

## 5 CONCLUSION

When women with PNDA are identified early and are supported to access appropriate supports, they are likely to experience a significant decrease in their symptoms, resulting in improved long‐term outcomes for them and their families (Dennis & Hodnett, 2007; Hadfield & Wittkowski, 2017). To that end, MCHNs hold a significant amount of responsibility, and are very well‐placed, to not only identify women with PNDA but also facilitate their timely access to appropriate interventions and offer a safe space for women to share their feelings. Our findings demonstrate that MCHNs honour this responsibility and strive to support women to the best of their ability. However, MCHNs work within a complex system, entrenched with a host of challenges that at times impede their ability to offer supports that are responsive to the needs of women with PNDA. Our findings emphasise an urgent need for policy and service delivery frameworks that facilitate equity in access to appropriate mental health care, regardless of socioeconomic status, ethnicity or place of residence. They also emphasise the significance of an integrated approach to supporting women with PNDA, so that no single professional body, including GPs or MCHNs, bears the lion's share of responsibility for facilitating women's access to psychological interventions. Finally, while our findings largely relate to MCHNs’ experiences of supporting women with PNDA, it also highlights the many challenges that women with PNDA, particularly those who are most vulnerable, face in gaining access to timely and appropriate care.

## 6 RELEVANCE TO CLINICAL PRACTICE

Our study identified a number of recommendations, put forth by MCHNs, which would greatly enhance their ability to support women with PNDA. They included the ability to operate as part of an integrated, multidisciplinary team of professionals; to offer additional and extended appointments when required and the ability to refer women directly to mental health and other relevant services, such as mother‐baby units. There is an urgent need to address the systemic barriers identified in this study, which hinder MCHNs’ ability to consistently respond to the needs of women experiencing PNDA. Facilitating equitable access to all perinatal mental health services and interventions must be at the heart of all future policy, funding and service delivery frameworks. Finally, greater investment is needed to address the current shortage of mental health services and specialists in rural areas, as well as the lack of mental health support options for women from non‐English speaking backgrounds.

## CONFLICT OF INTEREST

The authors declare that they have no known conflict of interest, including any competing financial interests or personal relationships that could have appeared to influence the work reported in this paper.

## Supporting information

Supplementary Material

## Data Availability

The data that support the findings of this study are available upon reasonable request from the corresponding author. The data are not publicly available due to privacy and ethical restrictions.
